# An explorative qualitative study to determine the footwear needs of workers in standing environments

**DOI:** 10.1186/s13047-017-0223-4

**Published:** 2017-08-30

**Authors:** Jennifer Anderson, Anita E. Williams, Christopher Nester

**Affiliations:** Centre for Health Sciences Research, University of Salford, Salford, M5 4WT UK

**Keywords:** Occupational, Footwear, Shoes, Musculoskeletal, Interview, Injury, Workplace, Standing

## Abstract

**Background:**

Many work places require standing for prolonged periods of time and are potentially damaging to health, with links to musculoskeletal disorders and acute trauma from workplace accidents. Footwear provides the only interaction between the body and the ground and therefore a potential means to impact musculoskeletal disorders. However, there is very limited research into the necessary design and development of footwear based on both the physical environmental constraints and the personal preference of the workers. Therefore, the purpose of this study was to explore workers needs for footwear in the ‘standing’ workplace in relation to MSD, symptoms, comfort and design.

**Method:**

Semi-structured interviews were conducted with participants from demanding work environments that require standing for high proportions of the working day. Thematic analysis was used to analyse the results and gain an exploratory understanding into the footwear needs of these workers.

**Results:**

Interviews revealed the environmental demands and a very high percentage of musculoskeletal disorders, including day to day discomfort and chronic problems. It was identified that when designing work footwear for standing environments, the functionality of the shoe for the environment must be addressed, the sensations and symptoms of the workers taken into account to encourage adherence and the decision influencers should be met to encourage initial footwear choice. Meeting all these criteria could encourage the use of footwear with the correct safety features and comfort. Development of the correct footwear and increased education regarding foot health and footwear choice could help to reduce or improve the effect of the high number of musculoskeletal disorders repeatedly recorded in jobs that require prolonged periods of standing.

**Conclusion:**

This study provides a unique insight into the footwear needs of some workers in environments that require prolonged standing. This user based enquiry has provided information which is important to workplace footwear design.

## Background

The nature of work related tasks and the design of many work places, makes standing the primary occupational posture. At least 50% of the employed population are exposed to the risks associated with prolonged standing [1, 2]. Prolonged standing, defined as standing for 50% or more of the working day [3], is associated with multiple health issues including chronic venous insufficiency, preterm birth, carotid atherosclerosis and work related musculoskeletal disorders [4]. The standing work places discussed in this paper include those that are predominantly standing with minimal ambulation.

Back and lower limb musculoskeletal disorders (MSD) are particularly prevalent with risk of low back and lower extremity/ foot pain being increased 1.9 and 1.7 fold respectively in those who stand for at least half their time at work [5]. Nealy et al. [6] reported that approximately 50% of nurses suffer MSD of the foot, substantially more than the 17.4% in the general population [7]. Similarly in perioperative staff, 43% reported pain in their leg or feet, compared to 12% in the general population [8]. The majority of staff (91%) attributed the pain to their work. For the employer, MSD can be costly in terms of absence and decreased efficiency [4].

As footwear provides the only interface between the body and ground when standing, alterations in footwear have the ability to influence the forces acting through the body, posture and movement [9], as well as to provide necessary protection against foot trauma and slips. Indeed, differences in footwear designs have been shown to affect fatigue and discomfort [10, 11], muscle activation and pressure under the foot [12, 13] all of which are factors relating to MSD. Therefore, wearing the correct footwear at work has the potential to reduce the risk of MSD and acute trauma.

A recent review paper highlighted that despite the detrimental impact of prolonged standing on the body, there is a scarcity of information relating to potential solutions, particularly in terms of flooring and footwear [8]. To ensure that workers wear the most suitable footwear, it is necessary to design and develop products based on both the physical environmental constraints and the personal preference of the workers. The limited research into the requirements of footwear from a workers perspective, particularly in relation to musculoskeletal symptoms, comfort, and design provides a starting point for understanding footwear in the workplace. By better understanding the footwear needs of workers, manufacturers may be able to produce footwear that will meet the requirements of the people who wear them and the environments they are worn in. For employers, this understanding can ensure the most appropriate footwear is identified, thus meeting their duty of care and reducing the likelihood of civil action from employees. Consequently, this study aims for the first time to explore workers needs for footwear in the ‘standing’ workplace in relation to MSD, symptoms, comfort and design.

## Method

Following ethical approval (University of Salford), participants were recruited through purposive sampling in relation to two occupations where standing is predominant and environments ‘challenging’ (when compared to office workers for example). The recruitment criteria was workers who work in demanding environments that require standing for the majority of the day. Multiple kitchens and veterinary hospitals were approached and within those that agreed to participate (3 kitchens, 1 veterinary hospital) staff volunteered if they wanted to take part after reading the participant information sheet. A total of 14 participants were included (kitchen staff: 8 (male: 6, female: 2), veterinary hospital theatre staff: 6 (male: 2, female: 4)). The number of participants is similar to that seen in other studies focusing on in-depth perceptions of footwear on specific conditions [14–16]. Participants provided informed consent and data collection (semi-structured, individual interviews) took place at their place of work by the researcher (JA). The interview was recorded digitally with supplementary field notes.

The participant’s job role, weekly working hours, time in job and type of shoes worn were recorded. The questions were non-specific to allow participants to talk about what was most relevant to them, but included their experiences and ideas of good/bad footwear features. Prompts were given during the interview where necessary. A list of questions and prompts can be seen (Table 1).

**Table 1:** List of questions and prompts used

Questions	Prompt examples
Do you experience any aches/ pains during work?	Where exactly?
Do you experience any aches/pains after work?	Can you point that out to me?
Do you experience any problems with your feet?	Anywhere else?
	How bad is the pain?
	Can you explain that further?
	Can you describe the pain?
What are the good aspects of your current shoes?	What do you mean by…?
What are the bad aspects of your current shoes?	Can you expand on that?
	What about the [insert part of shoe]?
Describe your perfect shoe	What do you mean by…?
	What would the [insert part of shoe] be like?
How would the style of the shoe be?	What do you mean by...?
	Can you expand on that?
If a shoe was described as comfortable, what would this mean to you?	What do you mean by…?
	Can you expand on that?
If a shoe was described as supportive, what would this mean to you?	
If a shoe was described as cushioned, what would this mean to you?	

The words that are regularly used to describe work footwear from a manufacturer’s point of view such as ‘comfortable’, ‘supportive’ and ‘cushioned’ were explored with each participant in relation to meaning and importance.

All interviews were transcribed verbatim by the researcher (JA). Thematic analysis was conducted in line with that described by Attride-Stirling [17]. The results were reviewed by a second researcher (AW) in order to confirm and agree meaning and interpretation.

## Results

**Fig. 1:**
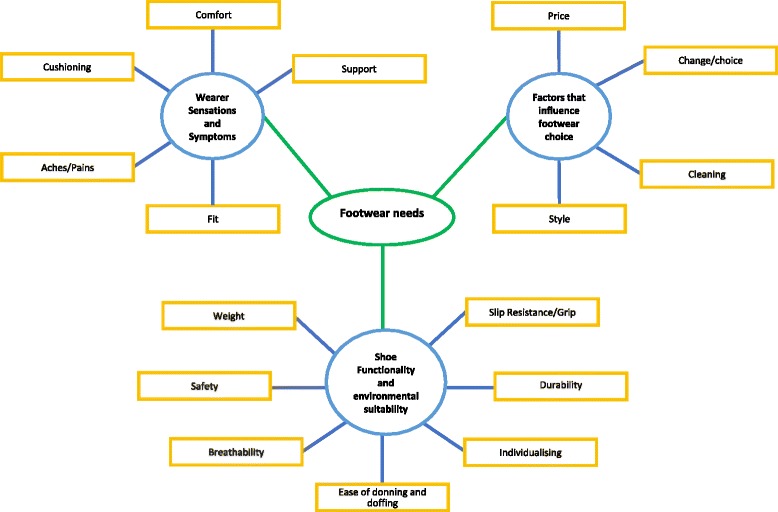
Thematic network comprising the global theme, organising themes and basic themes

Participant’s information was recorded at the beginning of the interview (Table 2). Basic and organising themes were grouped into a global theme of ‘footwear needs’ shown in Fig. 1. Table 3 displays the number of individuals that discussed each issue.

**Table 2:** Participant job and footwear information

Male/ Female	Job role	Years in job	Hours/week	Footwear description	Footwear make
Female	Commis Chef	1.5	50	Leather shoe with steel toe cap	Steel Lites
Male	Head chef	15	52.5	Clog, leather upper, cork footbed	Birkenstock
Male	Apprentice chef	1	50	Leather shoe with steel toe cap	-
Male	Sous chef	17	52.5	Leather clogs	Abeba
Male	Chef de Partie	2	52.5	PU clog (cork footbed)	Birkenstock
Female	Kitchen assistant	11.5	31.5	EVA clog	-
Male	Chef de Partie	1.5	50	Clog, synthetic upper	Dr. Brinkman
Male	Pastry chef	5	60	Clog, microfiber upper	Abeba
Female	Veterinary Surgeon	11	45	EVA clog	Toffeln
Male	Veterinary Opthalmologist	37	32	Chelsea boot	-
Female	Veterinary Nurse	32	37.5	EVA clog	Crocs
Female	Veterinary Nurse	3	37.5	EVA clog	Crocs
Male	Veterinary Surgeon	14	50	Leather upper clog	-
Female	Veterinary Nurse	3	38.5	Leather upper clog	Clarks

**Table 3:** Break down of themes with number of participant who mentioned them with or without prompting

	Mentioned without prompt	Mentioned with prompt	Not discussed
Shoe functionality			
Grip	11	2	1
Heat/ Breathability	10	4	0
Durability	9	0	5
Ease to don/doff	9	0	5
Fit	7	1	6
Safety	6	5	3
Weight	6	2	6
Individualising	3	0	11
Sensations and Symptoms			
Comfort	12	2	0
Support	6	8	0
Cushioned	5	9	0
Aches	4	10	0
Decision Influencers			
Cleaning	11	0	3
Price	10	0	4
Change/ choice	4	0	0
Style	3	9	2

Chefs worked an average of 50 ± 8 h a week and time in work ranged from 1 to 17 years (average = 7 years). Veterinary hospital staff worked on average 40 ± 6 h per week and time in work ranged from 3 to 37 years (average = 17 years). Both environments consisted of hard flooring throughout. The chefs prepared, cooked and presented food predominantly around kitchen counters/ cookers. Veterinary workers were based standing around operating tables during surgery but also undertook inpatient care and cleaning. In both environments, tasks varied based on individual roles. All individuals purchased their own footwear.

### Theme: wearer’s sensations and symptoms

The sensations and symptoms of the wearer whilst wearing the shoe can be broken down into five sub themes: aches and pains, comfort, cushioning, fit and support.

#### Aches/ pains

Four participants mentioned aches or pains at work without prompting, despite 13 of the 14 interviewees admitting to suffering some pain whilst at work once prompted. The attitude was that discomfort and/or aches were to be expected due to the job demands, and over time you grew accustomed to it. ‘After you’ve done it for quite a while you just sort of get on with it’, ‘I think I’m just used to it by now’ and ‘if you’re up doing stuff for that long …things will hurt’. Working long hours, standing and walking were all attributed to aches and pains ‘I’m standing for hours and hours, like 12 h days’, ‘after like a really long night…you really feel it, ‘walking around most of the time, and running up and down the stairs a lot’, ‘it’s just from standing I think’.

Both occupations described feeling discomfort or pain in the evening after work ‘After work as well. Especially if it’s a long day operating, then I’ll go home and be like bleurghhh’ and ‘at the end of a really long shift … everything will ache’ but also in the morning after a shift ‘when I wake up on a Sunday morning after a Saturday night I feel like I’ve been wearing heels all night’, ‘for about a year and a half getting out of bed in the morning, it would hurt’ and ‘if I’ve done a really, really long shift, the next day I feel it’. Some participants described not noticing the pain during the day, due to being particularly busy ‘I don’t notice it I’m just so busy’. Further, some describe working through the pain ‘you just get over it and carry on’, ‘After you’ve done it for quite a while you just sort of get on with it.’

Back pain was regularly mentioned with one chef stating ‘everyone else who works here has a lot of back pain problems’. In vets this was generally attributed to long hours standing, particularly ‘if I do a lot of surgery’. The ache from standing stationary and the ache after work were described as: ‘you just get that chronic ache that kind of builds up and then afterwards you just get that kind of dull ache’. The shin, calves, knees and feet were also areas described as aching ‘just like my calves and my feet ache’, ‘tends to be my feet and sometimes yeah in my legs actually’, ‘Knees, feet’, ‘knees sometimes’. One veterinary worker mentioned ‘I’ve got plantar fasciitis’ whilst two chefs complained of shin splints ‘I’ve had shin splints for probably... 5 years’. Being focused on the job was a key reason for not noticing aches or pains until after work ‘I don’t notice it I’m just so busy’. There was also a belief by some that aches and pains weren’t affected by the footwear ‘I think I could be wearing any shoes and it would still hurt’.

#### Comfort

The word ‘comfortable’ was used to describe the ideal shoe ‘it would have to be comfortable’. There was a need for both immediate comfort ‘Straight away they felt really comfy’ and long term comfort ‘Comfort over long hours is the main thing’. Further to this, participants were asked what the word comfortable meant to them. A lack of pain or discomfort was described ‘they don’t hurt you in any way ‘, ‘I guess like … not discomfort’ as well as not having to think about the shoe ‘it means that I don’t really notice them’. Comfortable shoes were also described as being able to ‘wear them anytime anywhere and you just don’t really mind because they’re comfy’.

#### Cushioning

Comfort definitions were strongly entwined to that of cushioning, with quotes including ‘cushions your foot’ and ‘comfortable makes you think of like a pillow’. Similarly, when asked to explain what the word cushioning meant, many participants mentioned the word comfort ‘Cushion is… even same as comfort’, ‘Just what gives it comfort’ and similarly a lack of pain ‘it won’t hurt when you put it on’. Again, comparisons to slippers and pillows were given ‘walking on pillows’. Suggestions that cushioning related to being ‘bouncy and springy’ were given ‘when you walk, you feel like you have a bounce’. One participant stated a cushioned shoe ‘conforms to your foot a bit more’.

Hard shoes were described negatively ‘It’s pretty hard it’s not comfortable’, ‘not very forgiving on your foot’ and ‘flat and hard and you can feel like the sole’. On the other hand, cushioned shoes were thought to ‘conform to your foot a bit more’ and also decrease the impact walking …‘the impact isn’t like as hard’. In chefs some described a negative relationship between cushioning and durability ‘the problem with like really cushioned shoes is it wears away pretty quickly’. One participant suggested ‘some bits of the sole need to be more cushioned than others perhaps… more cushioning in the heel’. Suggestions were made for a ‘middle ground’ between a hard and soft shoe.

#### Support

Numerous definitions were given to the word support in relation to footwear ‘like the ankle support’, ‘fitted and it would be enclosed’ and ‘supports the arch of the foot’. Support also had connotations to comfort ‘support is just like comfort’, ‘have to be comfortable so I suppose it has got be quite supportive of your foot’. The idea of spreading the foot pressure was also broached ‘if your arch is well supported, it kind of spreads the pressure better’. Finally, some suggested it would have a beneficial impact on the rest of the body ‘meant to stop you having back pains and leg pains’ and it ‘effects your whole posture’.

In terms of underfoot support, shoes with a flat footbed were negatively reviewed ‘If you’ve got a flat shoe it kind of puts a lot of pressure on the wrong bits of your foot’, ‘my trainers before were so painful because they were just like flat’. A preference was given to having arch support ‘I think they should have like all the support inside – like arch support and toe bits’, ‘it needs to support the flat part of my foot, like there needs to be a little arch in there’.

#### Fit

The main factor mentioned in relation to fit was that the footwear must remain on the foot. ‘It needs to fit. …needs to stay on my foot’ and ‘it needs to like fit well and my foot stays in the shoe’. Shoes that were loose on the foot were matched with a feeling of being unsafe ‘I didn’t feel safe in them because I thought they might just like fall off’. One method of keeping loose shoes on the foot was to ‘curl your toes a bit as well to keep them on’.

Two participants mentioned having wide feet as a problem ‘I’ve got wide feet so… they have to fit my feet’. One participant had problems purchasing the right size due to companies only selling whole sizes ‘because the sizes are not halves… I’ve got to get a 10 or 11 so have to wear an insole’.

### Theme: shoe functionality and environmental suitability

Shoe functionality and environmental suitability relates to any functions of the shoe and how they relate to the environment and job demands. It is comprised of 8 sub themes: grip, durability, safety, weight, breathability, ease of donning and doffing and individualisation.

#### Grip

Grip was a key factor, with 11 out of the 14 participants commenting unprompted on this theme. The high importance of it was emphasised in quotes, e.g. ‘that [slip resistance] is like probably at number one’, ‘the best ones have the best grip… the kitchen’s really slippery so you need grip’. Participants either described the underside grip on their shoe as being adequate ‘good treads – I’ve never skidded over in them’ or as being below their requirement ‘they don’t have much grip on the sole’. Some did not trust shoes marketed as slip resistant ‘… because a lot of shoes say they are [slip resistant] and they totally aren’t’. A veterinary worker suggested an issue with the front of the foot catching on the floor ‘I tend to catch the front of my toe and then I might go flying forward’.

Both vet and chef participants preferred to have grip on the inside of the shoe. ‘You need to have good grip inside them’, ‘if you get more grip inside it’s even better, even faster’. Whilst another stated ‘they’re easy to get wet and slippery on the inside’ as a negative about their current shoe.

#### Durability

Durability aligned with comments on grip, with suggestions that the grip wore out before any other footwear feature. This was supported by quotes such as ‘the grip doesn’t last very long’ and ‘the sole wears out really quickly’ and it was identified as a safety issue ‘The soles wear down really quickly and they just become like flat… which is really bad because you can just slip’. Grip durability was considered a point for future improvement ‘If there was any way that you could change how long the grip lasts’. Asides from grip, robustness of footwear as a whole was important. ‘They’re just good and durable’ was a positive whereas ‘they fell apart quite quickly’ and ‘they probably lasted less time than these would’ were negatives.

#### Safety

Toe protection was mentioned by chefs as a safety factor. Five (of 8) chefs thought safety toe caps should be used ‘I think that’s safer because I’m a bit clumsy – prone to dropping stuff’, ‘the safety bothers me, because anything can happen’ and ‘ideally it would have a toe cap’. However, the remaining three chefs did not feel a toe cap was necessary ‘I have never ever heard of someone to drop anything on their foot that’s going to like crush their foot’. This safety feature was of no concern to vets although one did state that ‘you’ve got to have toes covered so they don’t get trodden on’. Chefs identified further safety problems in their environments including knifes, pans and hot oil - ‘I’ve seen someone drop oil and it kinda melted the shoe into their foot’.

#### Weight

Weight was deemed an issue by chefs. ‘I don’t think they can be too heavy because it makes your day harder if you’ve got really heavy shoes on’ and ‘heavy shoes aren’t easy to walk around in’. In particular, some suggested heavy shoes became an issue as a result of constant moving ‘I don’t like anything heavy on my feet because I have to be up and down, up and standing… so heaviness will be a problem’ and ‘lighter is better... because here we do a lot of moving up and down, up and down’ One participant was particularly against heavy shoes ‘Heavy shoes is not an option for walking in the kitchen. No, I say no.’ A toe cap was considered heavy and excessive in weight, with one advocating it was not worth it ‘for the extra weight’.

#### Breathability

Heat and footwear breathability was considered to be a problem for both vets and chefs. Chef’s in particular described the environment as an issue to which increased breathability was a solution. ‘yeah breathable, it’s so hot in the kitchen anyway… it was like 35 degrees the other night…we were all dying it was so hot’, ‘It’s in the kitchen and the kitchen’s hot’ and ‘you need air in the kitchen because it’s warm’. Hot environments caused sweaty and odorous feet ‘my feet sweated a lot in those shoes and I used to get very itchy feet from that’, ‘make your feet smell… really bad’ and ‘I don’t like hot and sweaty feet’. Some participants removed their feet from their footwear to cool them ‘I can just take out my foot sometimes’ and ‘I quite often take my feet out’. A need for improved ventilation was recognised ‘maybe ones with some like breathing holes’ and ‘more ventilation would be quite nice’. An open back was also a positive as it would ‘be more airy’ and ‘quite good for keeping it cool’. However, both holes and an open backed shoe became a problem if the environment became wet: ‘if I had holes … I’d have soaking wet feet in 5 min’.

#### Ease of donning and doffing

An open backed shoe also linked to the theme of donning and doffing the shoes efficiently, which was mentioned by nine participants. It was a positive feature of current footwear ‘very easy to put on and take off’, ‘they’re convenient to put on as they just slip on’ and a requirement in the ideal footwear ‘something that’s easy to put on and take off’, ‘being able to slip them on… there’s no hassle’. Vets identified that shoes should be ‘easy to slip on and off so you can get into theatre’ but it was equally important for chefs ‘they’re convenient to put on as they just slip on’. Laces were seen as ‘a bit of a pain’ and it was easier not to ‘undo laces or flap around’. Fastenings of any kind were deemed negative by most ‘I’d definitely have like clog kind of things because I don’t really like lace ups or Velcro’. An open back was seen to increase the ‘the ease of getting them on and off quickly’.

#### Individualising

Individualisation of shoes in relation to fit and comfort was mentioned by 3 participants. ‘I think they have got to be tailored to you’. Different reasons for this were given. ‘Everyone has a different body, different feet…if you had like a foot analyst … and they worked out how we should have the shoes, like if people had low arches’. One proposed that this would reduce or eliminate the adjustment period to a pair of shoes ‘almost prescription... so you don’t have to let it mould to your foot’. Another stated that it would ‘make it more comfortable… if your shoe fits better, then it will lessen the chance of injury’.

### Theme: factors that influence footwear choice

These are the aspects that would influence the initial choosing of the footwear and can be broken down into four basic themes: cleaning, style, price and change/choice of footwear.

#### Cleaning

Cleaning was one of the most important factors relating to work footwear for both vets and chefs. Chef’s concern was ‘if you drop food on them’ whereas vets were worried about ‘blood, contamination’. The need for work footwear to be ‘easily cleanable’ and able to go in the washing machine were important factors. It was also important to be able to clean the inside of the shoe ‘the cleaning of the inside of the shoe…there’s odours you know’. Velcro or laces were a problem for chef’s ‘because if you drop food on them, it’s all in the laces and that’s just grim’ and also to food getting stuck in the grip on the bottom of the shoe ‘a real crucial one to me is the underside of the shoe…different shoes pick up different amounts of dirt’.

#### Style

The majority of individuals acknowledged that their work shoes and uniform were not attractive, ‘We always look fairly ridiculous’, ‘they make your feet look huge’ and ‘I wouldn’t wear them out [of work]’.However, all but one participant stated that the style was not of great importance with one chef stating ‘It doesn’t matter how it looks’ and a veterinary nurse similarly saying ‘look doesn’t really bother me’. Style was secondary to the function of the shoe: ‘I’m not too worried about the style, just about the comfort for me’ and ‘I’m more of a function over appearance’. Chefs and veterinary nurses outside of operating theatres expressed a preference towards black shoes ‘everyone wears black’ and ‘practice protocol is black shoes’ whereas inside the operating theatre the protocol was to wear white. ‘We tend to have white in theatre and other colours for out, just so you know the difference. So you know what’s clean and what’s not.’ One chef showed preference for a specific shoe brand ‘all the chefs in London had them’ and ‘they’re pretty trendy at the moment so I like them as a brand’ but also acknowledged ‘no one really cares that much’.

#### Price

Price was an essential factor in work footwear, with 10 participants mentioning it unprompted. When asked about their current footwear, one stated ‘I didn’t like the price’. There was a reluctance to spend money on new work shoes ‘cost… that’s why I haven’t gone out and bought any more’ and ‘you go through so many shoes, you don’t want to be spending so much money on a pair of shoes’. However, there was a trade off with price and durability with a willingness to spend more money on a pair of shoes if they were going to last and be of a higher quality. ‘It’s cost effective at the end of the day. If it’s going to last you know, twice as long as these, I’m happy with that’, ‘I’d probably spend a little bit more if I knew that they were going to last’ and ‘I would pay a bit more for a decent quality shoe’. Cheap shoes were described as inadequate ‘not made to your feet’ and ‘they skidded everywhere’.

#### Change/ choice of footwear

Some described having found a good shoe and wanting to stick with it ‘I just kept with them just because they fit my feet’ and ‘I’ve worn that sort of shoe for years and years’. Conversely one participant was unable to find the right shoe and described changing his shoes regularly ‘I got different shoe, different insoles so I got a lot of different shoes’ which was reinforced by another participant ‘It takes a good few years to work out what shoes actually work for you’. When choosing a shoe, there appeared to be a desire to fit in with everyone else and shoes were often purchased based on recommendations. ‘I just wore them because everybody else wore them’.

## Discussion

This is the first study to provide a unique insight into the footwear needs of workers in prolonged standing environments from a qualitative perspective. The footwear needs of vets and chefs can be broken into three key themes: sensations and symptoms of the worker; the function and suitability of the shoe for the environment and factors that influence footwear choice. Creating footwear that workers will adhere to wearing with the correct safety features and ergonomic design is a possible mechanism for injury prevention as it could improve safety [18] and reduce MSD [12, 19, 20]. Therefore this research has important implications for footwear design and manufacturing.

There was a high proportion of work related MSD reported (93%) that workers associated with the long hours on their feet. In agreement to previous studies that also found high rates of MSD in jobs requiring prolonged standing, the main areas affected were the back and lower extremities [5, 21, 22]. MSD were described as being obscured by occupational demands and participants identified a need to work through these aches and pains. This could cause conditions to develop and worsen as the summation of wear and tear from prolonged standing over time can result in chronic issues such as joint degeneration and chronic venous disease [4, 8, 23]. Furthermore, the reluctance to mention MSD in the workplace and the perception that they were an expected part of the job could reduce the chance of professional help being sought. Workers were affected both during and after work as well as the day following a long shift, signifying that quality of life outside of work could also be impacted.

Despite some beliefs that MSD are independent of the footwear worn, research indicates there is some potential to reduce aches, pains and feelings of fatigue through alterations in footwear or orthotic design [12, 19, 20, 24]. However, nothing specific to work place enviroments has been produced thus far. A few indications were made to the importance of footbed shape by relating flat footwear to an increase in pain and demonstrating a preference towards arch support. The literature supports this as a medial arch support increases the contact area and redistributes the plantar pressure of the foot [12]. However, due to the mix of beliefs regarding the link between MSD and footwear, educating workers on how different shoe features may impact on specific complaints could be required to avoid poor footwear choices, and this could include when to seek help from a health professional.

The work environments necessitate distinct footwear requirements. The specific flooring in both environments and high level of fluids result in a need for slip resistance. This was identified by almost all of the participants in this study as being of primary importance and has been demonstrated in previous studies to reduce slip rates by more than 50% [18]. Despite some misgivings about suitability of the slip properties of their current shoes, many participants still wore the footwear they deemed unsuitable. Due to the strong link between subjective and objective measures of friction [25], it is expected that use of a shoe that is perceived to have inadequate slip resistance would be detrimental to safety. Problems with the durability of the footwear grip was identified, and therefore it can be recommended that manufacturers should work to improve this or educate as to when footwear should be replaced. This is important for both safety and to align with criteria concerning generalised ‘durability’ and value of the footwear. Footwear that incorporated a method to identify when slip resistance reached an unsafe level could promote safety.

Heat is also an environmental concern for both vets and chefs. High temperatures were associated with hot, sweaty and odorous feet. High temperatures cause feet to sweat, creating a humid microclimate in the shoe, which results in discomfort [26] and exacerbates frictional forces that cause blisters [27]. Furthermore, sweat causes the surface of the skin to become more alkaline, promoting the development of pathogenic bacteria and fungi. As a solution to the discomfort, workers in both environments reported removing their feet from the footwear in order to cool them down, consequently exposing the foot to hazards. Therefore, it is clear that manufacturers must develop methods to maintain cooler in-shoe climates to improve comfort and reduce the risk of foot conditions developing.

The design of the shoe also influences the temperature, with an open back identified as much preferred due to the circulation of air it allows alongside the ease of donning/doffing the shoes. However, an open back and ventilation holes were unfavourable when the environment became wet. It is not always possible to create a perfect shoe for all environments and therefore features must be prioritised, or customised [28]. For these environments, allowing air into the shoe was the primary issue and therefore we would recommend prioritising the open backed shoe. However, feelings of being unsafe were promoted from shoes that did not remain on the foot. If the shoe did not hold the foot, workers had to resort to physical methods to hold the shoe on. Curling the toes whilst walking, a mechanism that is also adopted when wearing flip flops [29, 30], was used to hold the shoe in place. This could alter the way in which workers move as well as how the muscles are activating consequently impacting injury risk. A strap on an open backed shoe could improve the stability of the shoe on the foot whilst maintaining breathability.

Fit was an important footwear characteristic that was mentioned in its own right as well as in relation to comfort, donning and doffing of shoes and footwear individualisation. A good fitting shoe was given as a reason for not changing footwear, demonstrating its overall importance to footwear comfort and choice. Previously, it has been shown that fit is an important influencer of comfort, with other factors only influencing comfort when the fit was correct [31]. In particular, it was suggested that people with wide or narrow feet had issues purchasing good fitting footwear and there was a need for half sizes to improve the fit. Manufacturers can also play a role in guiding individuals to the correct size footwear, be it through online technology or in retail shops.

Initial and lasting comfort are both essential in work footwear. There is a similar high priority of footwear comfort for mail delivery, construction and care home workers with some workers choosing comfortable footwear over that with the correct safety features [28]. Comfort is related to the footwear, the task or activity and the characteristics of the individual worker such as skeletal alignment [32–35]. This highlights a potential requirement of different footwear for different occupations and reinforces that one shoe will not fit all. Comfort had positive associations with support, cushioning and the idea of footwear individualisation. Individualisation of the footwear or footbed shape was proposed to improve comfort and reduce injury. The literature reinforces that footwear customisation can enhance fit, comfort and prevent injury [36]. Whilst mass customisation would be extremely costly, there could be the option of using a best-matched fit method in which several options are made available and the individual worker chooses the most suitable. This could either be done for the whole shoe or just the footbed or insole and could be a cost-effective way to enhance comfort, meet customer desires [37] and perhaps reduce MSD.

There are a number of factors that can also influence footwear choice. It must be easy to clean and therefore have no fastening on the top that dirt can stick to. There is also a reluctance to spend money on work shoes and indeed it has previously been highlighted that leisure footwear is given higher financial priority than work footwear [28]. This study identified a price-quality trade off in which more money would be spent on a product if it was durable and of high quality. The perception of the factors involved in this trade off could be fundamental in terms of communicating the features of footwear, its benefits, and how this value proposition is proportional to price. Style is a secondary concern to the shoe function and comfort. This differentiates the needs of work footwear from that of leisure wear, where it has been suggested that style is preferential to comfort [38] and provides manufacturers an element of leeway in the shoe design. Chefs were more concerned with shoe appearance than vets, with mention of desirable brands. In these environments, general protocol dictates white or black shoes. It is also worth noting that the visual appearance of the shoe conveys perceptions about the shoe, including that relating to its function, performance and ergonomic quality, which could affect the purchase of the product [39, 40]. In this manner, footwear can be designed to match the consumers perceived needs and thus increase the chance of a worker choosing the shoe.

User preferences for work footwear and concerns regarding work related MSD have been largely ignored and this is the first study that we are aware of that focuses on user preferences for footwear in prolonged standing environments. Therefore this research is novel and provides a starting point from which the wider issues can be investigated. The use of open questions allowed identification of topic areas that were important to the participants. Using a small study sample of 14 participants decreases the generalisability of the results although this was not the aim of the study and the study aim of gaining an in-depth understanding from a few was met. Further, respondent bias could result from the self-volunteering nature of participant selection. The outcome that some preferences are work environment specific means that other environments might require separate investigation. The mixed group of participants from different environments in this study could also be a limitation, although they both met the purposive sampling criteria of standing for prolonged periods. In the future, a larger study could be used to investigate any differences between the two groups and to quantify any relationship between footwear and MSD..

## Conclusion

When designing the ideal work footwear for standing environments, the functionality of the shoe for the environment must be addressed, the sensations and symptoms of the workers taken into account to encourage adherence and the decision influencers should be met to encourage initial footwear choice. If any of these criteria are not met, workers are forced to choose based on favoured criteria, which can result in a decrease in safety features, comfort or both and could potentially lead to MSD or injury. Health professionals should take this into account when prescribing footwear or orthotics and footwear manufacturers must aim to meet all criteria. Future research is necessary to understand the link between footwear choice, work demands and MSD. The correct footwear and education regarding foot health and footwear choice could improve working conditions for workers and perhaps impact the high number of MSD repeatedly recorded in jobs that require prolonged periods of standing.

## Abbreviation

MSD: Musculoskeletal disorders

## References

[1] O’Neill R (2005). Standing problem. Hazards magazine.

[2] Parent-Thirion A, Vermeylen G, van Houten G, Lyly-Yrjänäinen M, Biletta I, Cabrita J (2012). Fifth European working conditions survey. Dublin. European Foundation for the Improvement of Living Working Conditions Ireland.

[3] Tomei F, Baccolo TP, Tomao E, Palmi S, Rosati MV (1999). Chronic venous disorders and occupation. Am J Ind Med.

[4] Halim I, Omar AR (2011). A review on health effects associated with prolonged standing in the industrial workplace. International Journal of Research and Reviews in Applied Science.

[5] Andersen JH, Haahr JP, Frost P (2007). Risk factors for more severe regional musculoskeletal symptoms: a 2 year prospective study of a general working population. Arthritis Rheum.

[6] Nealy R, McCaskill C, Conaway MR, Burns SM. The aching feet of nurses - an exploratory study. Medsurg Nurs. 2012;21 23477028

[7] Hill CL, Gill TK, Menz HB, Taylor AW (2008). Prevalence and correlates of foot pain in a population-based study: the North West Adelaide health study. J Foot Ankle Res.

[8] Meijsen P, Knibbe HJ (2007). Work-related musculoskeletal disorders of perioperative personnel in the Netherlands. AORN J.

[9] Anderson J, Williams AE, Nester CJ: A narrative review of musculoskeletal problems of the lower extremity and back associated with the interface between occupational tasks, feet, footwear and flooring. Musculoskeletal Care 2016; doi:10.1002/msc.1174. 10.1002/msc.1174 28032439

[10] Lin CL, Wang MJ, Drury CG (2007). Biomechanical, physiological and psychophysical evaluations of clean room boots. Ergonomics.

[11] Orlando AR, King PM (2004). Relationship of demographic variables on perception of fatigue and discomfort following prolonged standing under various flooring conditions. J Occup Rehabil.

[12] Chiu MC, Wang MJ (2007). Professional footwear evaluation for clinical nurses. Appl Ergon.

[13] Kersting UG, Janshen L, Bohm H, Morey-Klapsing GM, Bruggemann GP (2005). Modulation of mechanical and muscular load by footwear during catering. Ergonomics.

[14] Naidoo S, Anderson S, Mills J, Parsons S, Breeden S, Bevan E, Edwards C, Otter S (2011). “I could cry, the amount of shoes I can’t get into”:A qualitative exploration of the factors that influence retail footwear selection in women with rheumatoid arthritis. Journal of Foot and Ankle Research.

[15] Williams AE, Nester CJ, Ravey MI (2007). Rheumatoid arthritis patients’ experiences of wearing therapeutic footwear - A qualitative investigation. BMC Musculoskelet Disord.

[16] Paton JS, Roberts A, Bruce GK, Marsden J (2014). Patients’ Experience of therapeutic footwear whilst living at risk of neuropathic diabetic foot ulceration: an interpretative phenomenological analysis (IPA). Journal of foot and ankle research.

[17] Attride-Stirling J (2016). Thematic networks: an analytic tool for qualitative research. Qual Res.

[18] Verma SK, Chang WR, Courtney TK, Lombardi DA, Huang Y-H, Brennan MJ, Mittleman MA, Ware JH, Perry MJ (2011). A prospective study of floor surface, shoes, floor cleaning and slipping in US limited-service restaurant workers. Occup Environ Med.

[19] Gell N, Werner RA, Hartigan A, Wiggermann N, Keyserling WM (2011). Risk factors for lower extremity fatigue among assembly plant workers. Am J Ind Med.

[20] King PM (2002). A comparison of the effects of floor mats and shoe in-soles on standing fatigue. Appl Ergon.

[21] Messing K, Tissot F, Stock SR (2006). Lower limb pain, standing, sitting and walking: the importance of freedom to adjust one’s posture. Proceedings of the 16th Congress of the International Ergonomics Association.

[22] Sterud T, Tynes T (2013). Work-related psychosocial and mechanical risk factors for low back pain: a 3-year follow-up study of the general working population in Norway. Occup Environ Med.

[23] Bergan JJ, Schmid-Schönbein GW, Smith PDC, Nicolaides AN, Boisseau MR, Eklof B. Chronic venous disease. N Engl J Med. 2006:488–98. 10.1056/NEJMra055289 16885552

[24] Cambron JA, Duarte M, Dexheimer J, Solecki T (2011). Shoe orthotics for the treatment of chronic low back pain: a randomized controlled pilot study. J Manip Physiol Ther.

[25] Morio C, Bourrelly A, Sissler L, Gueguen N (2017). Perceiving slipperiness and grip: A meaningful relationship of the shoe-ground interface. Gait & Posture.

[26] Irzmańska E, Dutkiewicz JK, Irzmański R (2014). New approach to assessing comfort of use of protective footwear with a textile liner and its impact on foot physiology. Text Res J.

[27] Reynolds K, Darrigrand A, Roberts D, Knapik J, Pollard J, Duplantis K, Jones B (1995). Effects of an antiperspirant with emollients on foot-sweat accumulation and blister formation while walking in the heat. J Am Acad Dermatol.

[28] Norlander A, Miller M, Gard G. Perceived risks for slipping and falling at work during wintertime and criteria for a slip-resistant winter shoe among Swedish outdoor workers. Safety science. 2015;73:52–61.

[29] Price C, Graham-Smith P, Jones R (2013). A comparison of plantar pressures in a standard flip-flop and a FitFlop using bespoke pressure insoles. Footwear Science.

[30] Zhang X, Paquette MR, Zhang S (2013). A comparison of gait biomechanics of flip-flops, sandals, barefoot and shoes. Journal of foot and ankle research.

[31] Miller JE, Nigg BM, Liu W, Stefanyshyn DJ (2000). Influence of foot, leg and shoe characteristics on subjective comfort. Foot Ankle Int.

[32] Alemany S, González JC, García AC, Olaso J, Montero J, Chirivella C, Prat J, Sánchez J (2005). A novel approach to define customized functional design solution from user information.

[33] Goonetilleke RS (2001). Designing for Comfort: A Footwear Application.

[34] Miller JE, Nigg BM, Liu W, Stefanyshyn DJ, Nurse MA (2000). Influence of Foot, Leg and Shoe Characteristics on Subjective Comfort. Foot Ankle Int.

[35] Mündermann A, Stefanyshyn DJ, Nigg BM (2001). Relationship between footwear comfort of shoe inserts and anthropometric and sensory factors. Med Sci Sports Exerc.

[36] Salles AS, Gyi DE (2013). Delivering personalised insoles to the high street using additive manufacturing. Int J Comput Integr Manuf.

[37] Wang C, Tseng M. Mass customisation and footwear. In: Goonetilleke RS, editor The Science of Footwear. Boca Raton: CRC Press; 2012;625–642.

[38] Franciosa P, Gerbino S, Lanzotti A, Silvestri L (2013). Improving comfort of shoe sole through experiments based on CAD-FEM modeling. Med Eng Phys.

[39] Crilly N, Moultrie J, Clarkson PJ (2004). Seeing things: consumer response to the visual domain in product design. Des Stud.

[40] Riddle DL, Pulisic M, Pidcoe P, Johnson RE (2003). Risk Factors for Plantar Fasciitis: A Matched Case-Control Study. J Bone Joint Surg.

